# Gut Microbiota and Their Derived Metabolites, a Search for Potential Targets to Limit Accumulation of Protein-Bound Uremic Toxins in Chronic Kidney Disease

**DOI:** 10.3390/toxins13110809

**Published:** 2021-11-17

**Authors:** Mieke Steenbeke, Sophie Valkenburg, Tessa Gryp, Wim Van Biesen, Joris R. Delanghe, Marijn M. Speeckaert, Griet Glorieux

**Affiliations:** 1 Department of Internal Medicine and Pediatrics, Nephrology Unit, Ghent University Hospital, 9000 Ghent, Belgium; Mieke.Steenbeke@uzgent.be (M.S.); sophie.valkenburg@ugent.be (S.V.); tessa.gryp@poulpharm.be (T.G.); wim.vanbiesen@uzgent.be (W.V.B.); marijn.speeckaert@ugent.be (M.M.S.); 2 Laboratory Bacteriology Research, Department of Diagnostic Sciences, Ghent University, 9000 Ghent, Belgium; 3 Department of Diagnostic Sciences, Ghent University, 9000 Ghent, Belgium; joris.delanghe@ugent.be; 4 Research Foundation Flanders, 1000 Brussels, Belgium

**Keywords:** chronic kidney disease, uremic toxins, short-chain fatty acids (SCFAs), albumin symmetry factor, fecal samples

## Abstract

Chronic kidney disease (CKD) is characterized by gut dysbiosis with a decrease in short-chain fatty acid (SCFA)-producing bacteria. Levels of protein-bound uremic toxins (PBUTs) and post-translational modifications (PTMs) of albumin increase with CKD, both risk factors for cardiovascular morbidity and mortality. The relationship between fecal metabolites and plasma concentrations of PBUTs in different stages of CKD (*n* = 103) was explored. Estimated GFR tends to correlate with fecal butyric acid (BA) concentrations (*r_s_* = 0.212; *p* = 0.032), which, in its turn, correlates with the abundance of SCFA-producing bacteria. Specific SCFAs correlate with concentrations of PBUT precursors in feces. Fecal levels of *p*-cresol correlate with its derived plasma UTs (*p*-cresyl sulfate: *r_s_* = 0.342, *p* < 0.001; *p*-cresyl glucuronide: *r_s_* = 0.268, *p* = 0.006), whereas an association was found between fecal and plasma levels of indole acetic acid (*r_s_* = 0.306; *p* = 0.002). Finally, the albumin symmetry factor correlates positively with eGFR (*r_s_* = 0.274; *p* = 0.005). The decreased abundance of SCFA-producing gut bacteria in parallel with the fecal concentration of BA and indole could compromise the intestinal barrier function in CKD. It is currently not known if this contributes to increased plasma levels of PBUTs, potentially playing a role in the PTMs of albumin. Further evaluation of SCFA-producing bacteria and SCFAs as potential targets to restore both gut dysbiosis and uremia is needed.

## 1. Introduction

Chronic kidney disease (CKD) is characterized by a gradual loss of the kidney function, resulting in a decreased glomerular filtration rate (GFR) and urinary excretion of metabolites. Irreversible loss of the kidney function leads to a buildup of uremic waste products in the circulation in more advanced stages of CKD [1]. At uremic concentrations, these solutes have detrimental effects on different biological functions [2]. In recent years, a link between CKD and the gut microbiome has become apparent [3,4].

Important members of protein-bound uremic toxins (PBUTs) originate in the gut as a result of the bacterial metabolism [5]. In the distal part of the colon, tyrosine and phenylalanine are converted into *p*-cresol (*p*C), whereas tryptophan is converted into indole and indole acetic acid (IAA) [6]. These toxin precursors are absorbed and transported by intestinal epithelial cells [7]. During this transport, a small part of the precursors is already processed in the colonic mucosa to form the derived uremic toxins [8]. However, the majority of the precursors are processed in the liver [9], whereafter uremic toxins enter the circulation [10]. Mainly due to the decrease in the kidney function, their plasma levels rise in CKD [6]. CKD is also characterized by changes in the presence of intestinal bacterial species and a disruption of the intestinal barrier function [7,11]. 

There are several ways in which CKD can affect composition and functionality of the gut microbiome, such as the increased concentrations of urea, restrictive diets and the use of medications and antibiotics [3,12,13]. 

Short-chain fatty acids (SCFAs) are bacterial fermentation end products of dietary fibers, of which acetic acid (AA), propionic acid (PA) and butyric acid (BA) are the most common [14]. Species that are known to generate SCFA, in particular BA, are *Butyricicoccus* spp. [15], *Faecalibacterium prausnitzii* [16] and *Roseburia* spp. [17]. Besides being used as a source of energy by the host, SCFAs have a biological role in regulating the pH of the intestinal lumen, maintaining integrity of the intestinal barrier and regulating the host’s metabolism and immune response [18,19]. Aberrant levels of SCFAs are therefore linked with a variety of diseases [20]. A decrease in the production of SCFAs in the intestines is suggested to lead to an impaired intestinal barrier function [18]. Amino acids have been found to have similar beneficial effects on the intestinal barrier function by reducing oxidative stress and expression of proinflammatory cytokines [21]. This is characterized by a decrease in the expression of tight junction proteins and mucin, resulting in increased circulating levels of lipopolysaccharide (LPS) and insulin resistance. Gonzalez et al. demonstrated that the sole administration of BA to CKD mice decreased circulating LPS and restored insulin resistance [22]. 

The aim of this study was to explore the relationship between the abundance of SCFA-producing gut microbiota, fecal levels of SCFAs, fecal precursor levels of PBUTs and plasma concentrations of PBUTs in different stages of CKD.

## 2. Results

### 2.1. Fecal Levels of Short-Chain Fatty Acids over the Different Stages of CKD

**Figure 1:**
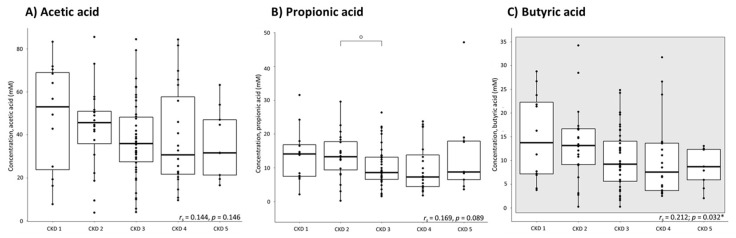
Fecal short-chain fatty acid concentrations per stage of chronic kidney disease (CKD): (**A**) acetic acid; (**B**) propionic acid; (**C**) butyric acid; *n* = 103 (CKD1: *n* = 12; CKD2: *n* = 20; CKD3: *n* = 42; CKD4: *n* = 20; CKD5: *n* = 9). Spearman’s rank test, the square indicates the significant correlation between the fecal butyric acid concentrations and the estimated glomerular filtration rate. Mann–Whitney test: o: *p* = 0.047 versus CKD 2; * significance is lost after the Bonferroni correction.

Fecal levels of AA, PA and BA were quantified at different stages of CKD (Figure 1). Fecal levels of BA especially tend to decrease with a declining eGFR (*r_s_* = 0.212; *p* = 0.032*), and a similar trend was observed for AA and PA (*r_s_* = 0.144, *p* = 0.146 and *r_s_* = 0.169, *p* = 0.089, respectively). A wide interpatient variability in fecal levels of SCFAs was observed. Multiple regression analysis indicated fecal abundance of *Roseburia* and fecal levels of indole to be the most important contributing factors to the variability in fecal levels of BA.

### 2.2. Association between the Presence of Short-Chain Fatty Acid-Producing Bacterial Species and Fecal Levels of Short-Chain Fatty Acids in CKD

**Figure 2:**
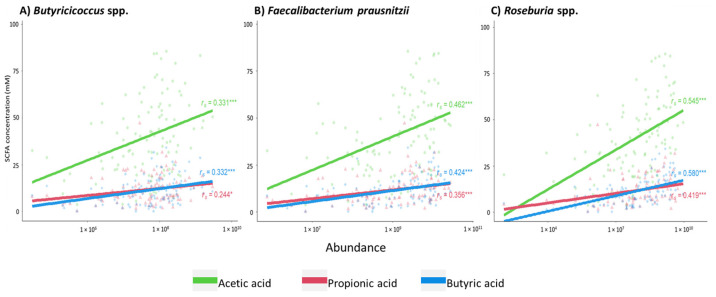
Correlation between the abundances of short-chain fatty acid (SCFA)-generating bacterial species and fecal SCFA concentrations: (**A**) *Butyricicoccus* spp.; (**B**) *Faecalibacterium prausnitzii*; (**C**) *Roseburia* spp. Acetic acid in green; propionic acid in red; butyric acid in blue. Correlations were calculated with Spearman’s rank test, *n* = 103. Significance of correlation is indicated: *** *p* < 0.001 and * *p* < 0.017 (level of significance after Bonferroni correction).

In this study, the positive correlation between eGFR and *Butyricicoccus* spp. and *Roseburia* spp. (*r_s_* = 0.260, *p* = 0.008 and *r_s_* = 0.287, *p =* 0.003, respectively) reported by Gryp et al. [11] was confirmed. A decrease in the amount of the SCFA-producing bacterial species *Butyricicoccus* spp., *Faecalibacterium prausnitzii* and *Roseburia* spp. was associated with a decrease in fecal levels of AA (*r_s_* = 0.331, *r_s_* = 0.462 and *r_s_* = 0.545, respectively; *p* < 0.001 for all the three bacterial species), PA (*r_s_* = 0.244, *p* = 0.013; *r_s_* = 0.356, *p* < 0.001; *r_s_* = 0.419, *p* < 0.001, respectively) and BA (*r_s_* = 0.332, *r_s_* = 0.424 and *r_s_* = 0.580, respectively; *p* < 0.001 for all the three bacterial species), as shown in Figure 2.

### 2.3. Correlation between Fecal Levels of Short-Chain Fatty Acids and Metabolites of the Amino Acid Metabolism

As precursors of plasma indoxyl sulfate (IxS), fecal tryptophan (Trp) and indole levels correlated positively with fecal levels of AA (*r_s_* = 0.349, *p* < 0.001 and *r_s_* = 0.285, *p* = 0.004, respectively), PA (*r_s_* = 0.326, *p* = 0.001 and *r_s_* = 0.377, *p* < 0.001, respectively) and BA (*r_s_* = 0.345, *p* < 0.001 and *r_s_* = 0.401, *p* < 0.001, respectively). For the amino acid-derived precursors of *p*-cresyl sulfate (*p*CS) and *p*-cresyl glucuronide (*p*CG), no significant correlation was found between SCFAs and fecal *p*C, tyrosine (Tyr) and phenylalanine (Phe).

### 2.4. Relationship between the Uremic Toxin Precursors in Feces and Their Corresponding Toxins in Plasma

Fecal levels of *p*C correlated with plasma levels of *p*CS (*r_s_* = 0.342; *p* < 0.001) and *p*CG (*r_s_* = 0.268; *p* = 0.006). In addition, a positive correlation was found between fecal levels of IAA and plasma IAA (*r_s_* = 0.306; *p* = 0.002). No significant correlation was found between fecal indole and plasma levels of IxS.

### 2.5. Fecal Levels of Short-Chain Fatty Acids Are Associated with Plasma Levels of p-Cresol-Derived Uremic Toxins, p-Cresyl Sulfate and p-Cresyl Glucuronide

Plasma levels of *p*CS and *p*CG correlated negatively with fecal levels of SCFAs: AA (*r_s_* = −0.319, *p* = 0.001 and *r_s_* = −0.344, *p* < 0.001, respectively), PA (*r_s_* = −0.289, *p* = 0.003, and *r_s_* = −0.297, *p* = 0.002, respectively) and BA (*r_s_* = −0.423, *p* < 0.001 and *r_s_* = −0.401, *p* < 0.001, respectively). In contrast, plasma levels of IxS and IAA were not significantly correlated to fecal levels of any of the SCFAs.

### 2.6. Albumin Modifications in CKD

**Figure 3:**
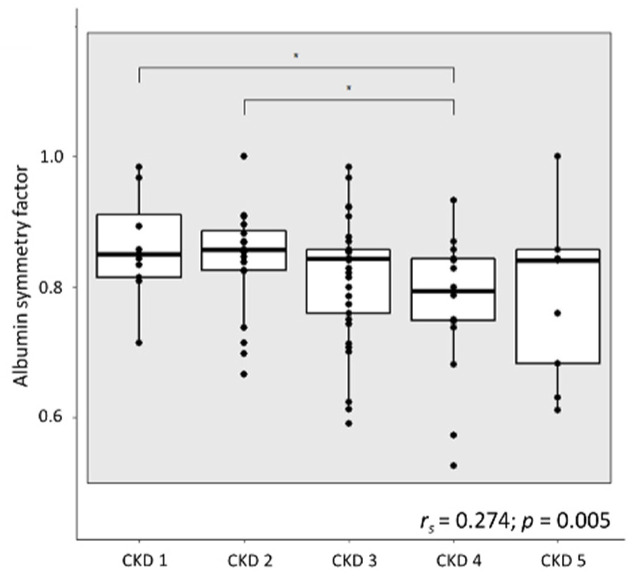
Albumin symmetry factor (ASF) per stage of chronic kidney disease (CKD) (*n* = 103; CKD1: *n* = 12; CKD2: *n* = 20; CKD3: *n* = 42; CKD4: *n* = 20; CKD5: *n* = 9; * *p* < 0.05); Spearman′s rank test with the estimated glomerular filtration rate (eGFR) as a continuous variable, square indicates the significant correlation between ASF and eGFR.

The albumin symmetry factor (ASF) was calculated from the spectra of capillary electrophoresis in different stages of CKD. Overall, a decrease of ASF, a predictor of the degree of albumin post-translational modifications (PTMs), was observed with a decrease in the kidney function (*r_s_* = 0.274; *p* = 0.005 for eGFR as a continuous variable), as illustrated in Figure 3. Albumin PTMs were the most pronounced in plasma samples from patients in CKD stage 4 versus CKD stage 1 (*p* = 0.026) and CKD stage 2 (*p* = 0.023), respectively (Figure 3).

### 2.7. Plasma Levels of and Protein-Bound Uremic Toxins and Percentage of Protein Binding Are Associated with the Albumin Symmetry Factor

Increased plasma levels of *p*CS, *p*CG and IxS were negatively correlated with ASF (*r_s_* = −0.274, *p* = 0.005; *r_s_* = −0.304; *p* = 0.002; *r_s_* = −0.268, *p* = 0.006, respectively). No significant correlation was found for plasma levels of IAA. In addition, no correlation was found between the abundance of SCFA-producing bacteria or fecal levels of SCFAs and ASF. A decrease in ASF, or increase in PTMs, was associated with a decrease in the percentage of protein binding of the indole-derived uremic toxins IxS and IAA (*r_s_* = 0.432, *p* < 0.001 and *r_s_* = 0.339, *p* < 0.001 respectively).

## 3. Discussion

CKD is characterized by increased morbidity and mortality, which is, at least in part, attributed to uremic toxicity [23]. Several of the associated uremic toxins originate in the intestines by bacterial metabolization of dietary products. Some of these intestinally generated uremic toxins are bound to proteins in the circulation, and their removal by dialysis is expensive and remains insufficient [24,25]. Alternative measures to decrease levels of these uremic toxins, preferably targeting uremic toxins at their origin, are needed.

**Figure 4:**
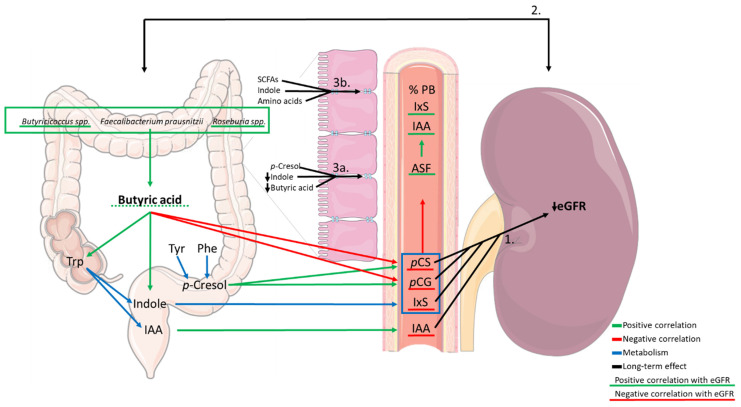
Summary of the association between short-chain fatty acid (SCFA)-producing bacteria, SCFAs (in casu butyric acid) and amino acids and protein-bound uremic toxin (PBUT) precursors in feces, plasma levels of PBUTs and the albumin symmetry factor (ASF) and the estimated glomerular filtration rate (eGFR). Green arrows indicate a positive correlation, red arrows indicate a negative correlation, blue arrows indicate metabolism, black arrows indicate a long-term effect. Underlined factors are correlated to eGFR (green underlining: positive correlation with eGFR; red underlining: negative correlation with eGFR; the dotted line indicates the loss of significance after the Bonferroni correction). While the decrease in the kidney function building up the uremic milieu (1) can influence the gut bacteria and their metabolism causing dysbiosis, this dysbiosis can further contribute to the loss of the kidney function, as indicated by the bidirectional arrow (2). Zoom image: In CKD, next to the decrease in fecal levels of BA [18], the decrease in fecal levels of indole and the presence of *p*-cresol can have deleterious effects on the intestinal barrier function [28,29] by decreasing expression of tight junction proteins and thus increasing the paracellular transport which can contribute to increased plasma levels of PBUTs (3a). In healthy intestines, SCFAs, indole and amino acids strengthen functionality of the intestinal barrier (3b).Trp: tryptophan; Tyr: tyrosine; Phe: phenylalanine; IAA: indole acetic acid; PB: protein binding; *p*CS: *p*-cresyl sulfate; *p*CG: *p*-cresyl glucuronide; IxS: indoxyl sulfate.

Furthermore, levels of SCFAs have been associated with cardiovascular disease in CKD [26]. The present data show that, in a group of CKD patients not on dialysis, fecal levels of BA tend to be associated with kidney function (eGFR), which is in line with the findings of Wang et al. [27]. SCFA-producing gut bacteria *Faecalibacterium prausnitzii*, *Roseburia* spp. and *Butyricicoccus* spp. correlated with fecal levels of SCFAs (AA, PA and BA) in patients with CKD. In addition, correlations between fecal concentrations of SCFAs and uremic toxin precursors and the plasma levels of intestinally generated uremic toxins were observed. In Figure 4, the most important findings are summarized for BA. 

The scheme shows three different compartments involved in the gut–kidney axis. A decrease in the abundance of SCFA-producing bacteria is correlated to a decrease in the concentration of fecal levels of SCFAs, especially of BA. In turn, decreased levels of BA are positively correlated to fecal levels of Trp and indole, precursors of IxS and IAA. Fecal levels of butyric acid are negatively correlated to plasma levels of *p*CS and *p*CG. Plasma levels of the PBUTs *p*CS, *p*CG and IxS were negatively correlated to ASF. An increase in PTMs of albumin can decrease the degree of binding of uremic toxins to albumin as reflected by the positive correlation of ASF with the percentage of protein binding of IxS and IAA.

Dysbiosis of the gut resulting in an overgrowth of pathobionts, a disruption of the intestinal barrier function and increased inflammation is common in CKD [30]. Based on their metabolism, bacteria can be categorized into saccharolytic and proteolytic bacteria [31,32]. Saccharolytic bacteria predominantly ferment carbohydrates into SCFAs, which are generally considered to be favorable compounds for health and function of the intestinal barrier [18,31]. SCFAs serve multiple purposes that together contribute to the integrity of the intestinal barrier. AA is the most abundant SCFA in the colon, which is mostly used as the energy source for the peripheral tissues, as the substrate for lipogenesis and cholesterol biosynthesis in the liver [33,34]. PA is mainly transported to the liver, where it serves the same main purposes as AA. BA is an important source of energy for colonocytes, with up to 70% of their needed energy derived from the metabolism of SCFAs. It regulates the pH in the colon, protects against harmful metabolites of bile acid and phenols, regulates enteroendocrine hormones and is assumed to be involved in the production of ketone bodies in the liver [19,34]. Decreased abundance of SCFA-producing bacteria [11,35] as well as fecal SCFAs levels [27,36] demonstrated in CKD and end-stage kidney disease are associated with intestinal inflammation, inflammatory bowel disease and colorectal cancer [37,38]. BA-producing capacity has been described previously for *Faecalibacterium prausnitzii* [16], *Roseburia* spp. [17] and *Butyricicoccus* spp. [15], while AA production was only described for *Butyricicoccus* spp., PA for none of the studied species. Although a significant positive correlation was found between the abundance of *Butyricicoccus* spp., *Faecalibacterium prausnitzii* and *Roseburia* spp. and fecal levels of the three SCFAs, AA-producing capacity for *Faecalibacterium prausnitzii* and *Roseburia* spp. and PA-producing capacity for all the three evaluated bacterial species was not proven. In fact, *Faecalibacterium prausnitzii* and *Roseburia* spp. are known to consume AA [39,40]. A potential explanation for this finding may lay in the coexistence of multiple specific species of bacteria in the intestinal environment, which is at the base of the cross-feeding principle as seen, e.g., between the genera *Bifidobacterium* and *Faecalibacterium* [41], in which the faecalibacteria feed on the AA produced by the bifidobacteria. 

Besides SCFAs, the gut microbiota also produces uremic toxins and their precursors. Fecal levels of the uremic toxin precursors Tyr, Phe, *p*C and Trp, indole and IAA were quantified. Unexpectedly, fecal levels of all the three SCFAs positively correlated with fecal levels of Trp and indole and were not associated with fecal levels of *p*C. While a decrease in SCFA generation in CKD is expected to be associated with a decrease in saccharolytic fermentation/bacteria, in parallel, an increase in the proteolytic fermentation/PBUT-producing bacteria is anticipated. Unfortunately, the existing data cannot distinguish between the two intestinal-related mechanisms that can contribute to the increase in plasma levels of PBUTs, (1) bacterial metabolite generation and (2) transport over the intestinal barrier, so further investigation on this issue is needed. It is of note that indole, like the previously described SCFAs, is a favorable compound for intestinal health, e.g., through the increase of epithelial cell tight junction resistance, control of factors of inflammation and protection of colitis [29,42]. A decrease in fecal indole on top of a decrease in fecal levels of SCFAs could further contribute to a disruption of the intestinal barrier function in CKD. In this way, increased paracellular transport of precursors of uremic toxins towards the circulation (so-called “leakage”) further enhances the increase in plasma levels as suggested by the correlation between fecal levels of *p*C and IAA and the plasma levels of their respective derivatives, *p*CS, *p*CG and IAA. While no clear link was found between the fecal levels of indole and plasma IxS, a potential shift in the Trp pathways (indole, serotonin and kynurenine) in CKD should be further explored.

SCFAs have a beneficial effect on the kidney function by improving kidney injury and delaying CKD progression [27,43]. It is, however, not clear what the mechanism is behind the improved kidney function, it might be the result of decreased levels of uremic toxins as demonstrated for TMAO [36]. Wang et al. also reported a decrease in fecal levels of BA and serum levels of SCFAs in CKD patients versus the control subjects [27]. However, no clear correlation between fecal and serum levels of SCFAs was demonstrated, which needs further evaluation. This could potentially provide more insights into the changes in uptake and use of SCFAs over different stages of CKD. 

In a previous evaluation by our group, no difference in fecal levels of uremic toxin precursors was observed over the different stages of CKD, and the rise in plasma levels of the intestinally generated uremic toxins was mainly attributed to the decrease in kidney (tubular) function [6]. In this evaluation, the negative correlation between fecal levels of all the three SCFAs and plasma levels of *p*CS and *p*CG suggests that, in addition to decreased kidney function, disruption of the intestinal barrier function by low fecal levels of beneficial compounds such as SCFAs and indole might affect plasma levels of *p*CS and *p*CG more than the degree of generation of its mother compound *p*C. It needs to be investigated whether restoration of the intestinal barrier function (by increasing SCFA levels) results in decreased plasma levels of *p*CS and *p*CG. 

The abundance of the SCFA-producing bacteria and the fecal levels of SCFAs were not associated with the marker of albumin modification, ASF, in CKD. ASF was found to be strongly correlated to eGFR, confirming the increased abundance of PTMs in the later stages of CKD [44]. A negative correlation of ASF to several measured uremic toxins, including total levels of *p*CS, *p*CG and IxS, indicates an effect of these uremic toxins, per se, on the ability of albumin to bind these uremic toxins. This is in line with multiple studies over recent years showing a link between CKD and PTMs through increased levels of uremic toxins, in particular urea [44,45,46]. The decrease of ASF with the progression of CKD is accompanied by a decrease in the degree of protein binding of IxS and IAA and could be translated to higher toxicity of these uremic toxins due to the increased free circulating fraction [2]. However, no significant correlation was found between ASF and the *p*C-derived toxins. 

It is important to note that in this study, only correlations and no causal effects were investigated and that the large interpatient variability in fecal levels of SCFAs and a relatively small sample size might have contributed to the lack of correlation in some cases. Nevertheless, the observed correlations indicate and justify further evaluation of potential causal effects of SCFAs. Another weakness of the study is that although disruption of the intestinal barrier function has been described repeatedly in CKD [22,47], no estimation of intactness of the intestinal barrier function was investigated in this study. Further exploration of the generation of and the balance between toxic and beneficial metabolites in the intestinal compartment is needed. In parallel, the effect of these metabolites on the intestinal barrier function and the active transport and/or leakage of metabolites as well as potential interventions to influence these processes needs in-depth investigation.

The decreased abundance of SCFA-producing gut bacteria in parallel to the fecal concentration of BA and indole can compromise the intestinal epithelial barrier function in CKD. It is currently not known whether the changes in fecal levels of SCFAs contribute to increased plasma levels of PBUTs. However, the obtained results justify further evaluation of fecal SCFA-producing bacteria and fecal SCFAs as potential targets to restore both gut dysbiosis and decrease the accumulation of intestinally generated uremic toxins.

## 4. Materials and Methods

### 4.1. Cohort

The study population from which fecal and plasma/serum samples were used was described previously [6]. In this study, 103 non-dialyzed CKD patients were included. The study group consisted of 12 patients in CKD stage 1, 20 patients in CKD stage 2, 42 patients in CKD stage 3, 20 patients in CKD stage 4 and 9 patients in CKD stage 5. The exclusion criteria were active infection (C-reactive protein (CRP) > 20 mg/L), immunosuppressive therapy, obesity (body mass index (BMI) > 35 kg/m^2^), inflammatory bowel disease, active malignancy, a cardiovascular event in the past three months, pregnancy, transplantation, use of nonsteroidal anti-inflammatory drugs within the past month and age < 18 years. Before inclusion, all the patients provided written informed consent. The study was conducted in accordance with the Declaration of Helsinki and approved by the Medial Ethics Committee of Ghent University Hospital (Ref. No. 2010/033, B67020107926).

### 4.2. Quantification of Bacterial Species in Fecal Samples

Quantification of select SCFA-producing bacterial species was previously described [11]. In brief, using a PowerMicrobiome RNA isolation kit (Qiagen, Hilden, Germany), DNA and RNA were extracted from the fecal samples. Family-, genus- and species-specific quantitative polymerase chain reactions (qPCRs) were developed and used [11]. All the samples were amplified in duplicate for each of the qPCR reactions, and a dilution series was used as the positive control; qPCR mixes without DNA served as the negative control. 

### 4.3. Preparation of the Fecal Suspension

Fecal suspensions were prepared as previously described [6]. In brief, anaerobic phosphate buffer was added to the fecal samples and vortexed. This fecal suspension was centrifuged and the supernatant was stored at −80 °C after filtration with a 0.22 µm filter to remove viable bacteria before ultraperformance liquid chromatography (UPLC) analysis.

### 4.4. Quantification of Uremic Toxins Precursors in the Fecal Suspension and Plasma of CKD Patients

Fecal levels of uremic toxin precursors and plasma levels of uremic toxins in the present cohort were reported by Gryp et al. [6]. In brief, the fecal suspensions, Tyr (181 Da), Phe (165 Da), Trp (204 Da), *p*C (108 Da), and indole (117 Da) were separated by high-performance liquid chromatography (HPLC) and detected using a Waters 2475 fluorescence detector (Waters, Milford, MA, USA) (Tyr (λex: 275 nm, λem: 302 nm), Phe (λex: 257 nm, λem: 282 nm), Trp (λex: 280 nm, λem: 348 nm), *p*C (λex: 278 nm, λem: 304 nm), indole (λex: 275 nm, λem: 334 nm) and fluorescein as the internal standard (λex: 443 nm, λem: 512 nm)). In the fecal suspension and plasma, the total and free concentrations of *p*CS (187 Da), *p*CG (284 Da), IxS (213 Da) and IAA (175 Da) were quantified (IxS (λex: 280 nm, λem: 376 nm), *p*CS and *p*CG (λex: 264 nm, λem: 290 nm), IAA (λex: 280 nm, λem: 350 nm) and fluorescein (λex: 443 nm, λem: 512 nm)) using a fluorescence detector after separation by UPLC (Agilent, Santa Clara, CA, USA)as described by Gryp et al. [6] and El Amouri et al. [48].

### 4.5. Quantification of Short-Chain Fatty Acids in the Fecal Suspension of CKD Patients

The method of quantification of AA, PA, and BA in the fecal suspension by ULPC and diode array detection (DAD) (Agilent) was validated. A detailed description can be found in the Supplementary Materials (S1; S2 and Tables S1 and S2). In brief, liquid–liquid extraction of the samples was performed. Chromatographic separation was performed on an XBridge BEH C18 XP column (150 mm × 4.6 mm internal diameter (i.d.)) with a particle size of 2.5 μm (Waters, Milford, MA, USA) protected by a guard column of the same type (5 mm × 3.9 mm i.d.). The DAD detector was set at the wavelength of 210 nm using the peak width of 5 Hz. Data processing was performed using Open Lab CDS ChemStation Edition for LC & LC/MS Systems Rev C.01.07.SR2 [255] (Agilent Technologies, Santa Clara, CA, USA). Levels of SCFAs were normalized for 1 g of feces.

### 4.6. Quantification of the Albumin Symmetry Factor

Blood samples were collected in serum separator tubes (Becton Dickinson, Franklin Lakes, NJ, USA). The blood samples were placed for 20 min at room temperature (RT) for coagulation, followed by a centrifugation step at 2095 g for 10 min at 4 °C. Serum was collected, aliquoted and stored at −80 °C till batch analysis.

ASF, a predictor of the degree of PTMs of albumin, was assayed on a capillary electrophoresis instrument (Helena V8) (Helena, Newcastle, UK) [49]. In brief, ASF has been defined as the distance from the centerline of the albumin peak to the back slope divided by the distance from the centerline of the albumin peak to the front slope, with all the measurements made at 10% of the maximum albumin peak height [49]. A lower ASF corresponds with a higher amount of PTMs of albumin.

### 4.7. Other Parameters

Serum creatinine (113 Da) was determined using the photometric isotope dilution mass spectrometry (ID-MS) calibrated alkaline picrate method (Architect c160000, Abbott, Chicago, IL, USA) in the routine laboratory of Ghent University Hospital, Belgium. The estimated glomerular filtration rate was calculated with the chronic kidney disease epidemiology collaboration (CKD-EPI) formula [50].

### 4.8. Statistical Analysis

All statistical analyses were performed using IBM SPSS Statistics for Windows, version 27 (IBM, Armonk, NY, USA), and the graphs were made with Rstudio (2020, version 4.0.2) (Rstudio, Boston, MA, USA). Before statistical analysis of the concentrations of precursors, toxins, SCFAs, and bacteria, the normality was tested using the Kolmogorov–Smirnov test. Based on the distribution of data, correlations were calculated using Spearman′s rank tests; in addition, regression analysis was performed for BA. For SCFAs and ASF, individual stages of CKD were compared to each other with the Mann–Whitney test for independent groups; *p* < 0.05 was considered statistically significant. However, in the case of multiple testing, the Bonferroni correction was applied in order to adjust the level of significance to *p* < 0.017 in the case of *n* = 3 and *p* < 0.0125 in the case of *n* = 4.

## Data Availability

All data are fully available without restriction in an anonymized format upon request.

## Supplementary Materials

The following are available online at https://www.mdpi.com/article/10.3390/toxins13110809/s1, S1: Supplementary Methods: Quantification of Short-Chain Fatty Acids in Fecal Suspension, S2: Supplementary Results: Quantification of Short-chain fatty Acids in fecal suspension, Table S1: Dilution scheme to obtain the calibrators, Table S2: Gradient programme for the analysis of short-chain fatty acids (SCFAs) in fecal water.
