# Supplementary material for: Gut Microbiota and Their Derived Metabolites, a Search for Potential Targets to Limit Accumulation of Protein-Bound Uremic Toxins in Chronic Kidney Disease
Source: Toxins (Basel). 2021 Nov 17;13(11):809. doi: 10.3390/toxins13110809 (PMC8625482; doi:10.3390/toxins13110809)
Supplement: Supplementary file 1 [file toxins-13-00809-s001.zip › toxins-1448756-supplementary.pdf]

Supplementary Materials: Gut Microbiota and Their Derived Metabolites, a Search for Potential Targets to Limit Accumulation of Protein-Bound Uremic Toxins in Chronic Kidney Disease

Mieke Steenbeke, Sophie Valkenburg, Tessa Gryp, Wim Van Biesen, Joris R. Delanghe, Marijn M. Speeckaert and Griet Glorieux

S1. Supplementary Methods: Quantification of Short-Chain Fatty Acids in Fecal Suspension

Standards, Chemicals, and Reagents

Acetic acid (AA, P/N 5.43808), propionic acid (PA, P/N 94425), butyric acid (BA, P/N 19215), and sodium succinate dibasic hexahydrate (P/N S2378) were obtained from Merck (Merck, Darmstadt, Germany) and stored at room temperature (RT).

Stock solutions of 5 M AA, PA and BA were prepared in HPLC grade water (Biosolve, Valkenswaard, Netherlands) and stored at 2-8°C for 3 months. For sodium succinate dibasic hexahydrate, which was used as internal standard (IS), a stock solution of 0.09 M was prepared.

The combined working solution (WS, containing AA, PA, and BA) of 100 mM was prepared by diluting the stock solution appropriately with phosphate-buffered saline (PBS) (P/N BE17-516F, Lonza, Verviers, Belgium). The 100 mM working solution was used to prepare the working solutions of 25, 10, 5, and 2.5 mM by diluting the 100 mM working solution appropriately with PBS (Lonza). The working solutions were prepared freshly each time a calibration curve was set up.

For the liquid-liquid extraction following reagents were needed: hydrochloric acid (HCl, 37%) (VWR, Leuven, Belgium), NaOH (Merck), and diethylether (Merck). For the ultra high performance liquid chromatography [U(H)PLC] analysis following chemicals were needed: HPLC grade water (Biosolve), methanol (Biosolve), and acetonitrile (Biosolve), and phosphoric acid (VWR).

Sample Preparation

Calibration Curve

A stock solution of 5 M AA, PA and BA in HPLC grade water was prepared. The working solution of 100 mM was prepared by adding 100 µL of the 5 M stock solution of each standard namely, AA, PA, BA and 4700 µL PBS. The 100 mM solution was used to prepare the other working solutions in PBS: 25 mM, 10 mM, 5 mM and 2.5 mM. The following calibrators were prepared: 0.25, 0.5, 1, 2.5, 5, 10, 15 and 25 mM and the dilution scheme is presented in Supplementray Table S1. Of each calibrator 400 µL was pipetted at RT into a 10 mL pyrex extraction tube.

Blank Sample

PBS (400µL) was pipetted at RT into a 10mL pyrex extraction tube.

Study and Control Samples

For the control sample a pool of six fecal suspension of healthy persons was prepared.

PBS (400 µL) at RT for the blank sample and 400 µL of the fecal suspension at 4°C for the control and study samples were pipetted into separate 10 mL pyrex extraction tubes.

Liquid-liquid Extraction

To each sample of an analytical batch (calibrators, control sample, study samples and blank sample) 20 µL of the IS stock solution (0.09 M sodium succinate dibasic hexahydrate) was added. The samples were vortex mixed for 5 seconds (sec). Thereafter 40 µL of concentrated HCl was added, followed by a vortex mixing step of 15 sec. The samples were extracted by adding 2 mL diethylether with the HandyStep pipet (Brandtech Scientific, Essex, CT, USA), vortex mixing for 5 sec, three times manually mixing the pyrex extraction tubes and placing the pyrex extraction tubes horizontally while

shaking at RT for 20 min. Before centrifugation (5 min, 2,851*g*, 20°C), the samples were manually mixed three times. After centrifugation, the supernatant was transferred to a second pyrex extraction tube and 200 µL of a 1 M NaOH solution was added. A second extraction was performed by vortex mixing the samples for 5 sec, manually mixing the pyrex extraction tubes three times and placing the pyrex extraction tubes horizontally, while shaking warm water bath at RT for 20 min. Before centrifugation (5 min, 2,851*g*, 20°C), the samples were again manually mixed three times. The aqueous phase (200 µL) was transferred to a 1.5 mL Eppendorf vial. Of the latter phase, 150 µL was transferred to an HPLC autosampler vial and 30 µL of concentrated HCL was added. After vortex mixing for 15 sec and removing air bubbles, 10 µL of the aliquot was injected.

Ultra-performance Liquid Chromatography Method

The UPLC system consisted of a G7104A quaternary pump with integrated degasser, a G4226A autosampler, a G1330B thermostat, a G1316C column compartment, and a G4212A diode-array detector (DAD), all from Agilent Technologies (Agilent Technologies, Santa Clara, CA, USA). The autosampler temperature was set at 8°C. Chromatographic separation was carried out on an XBridge BEH C18 XP column [150 mm x 4.6 mm internal diameter (i.d.)] with a particle size of 2.5 µm (Waters, Milford, MA, USA). The HPLC column was protected by a guard column of the same type (5 mm x 3.9 mm i.d.). The injection volume was 10 µL using a needle wash of 20% methanol in water for 3 sec. The column was thermostated at 30°C. The mobile phase (MP) consisted of 0.2% phosphoric acid in water (A), methanol (B), and acetonitrile (C). Gradient elution was performed as shown in Supplementary Table S2. The DAD detector was set at a wavelength of 210 nm using a peak width of 5 Hz. Data processing was performed using Open Lab CDS ChemStation Edition for LC & LC/MS Systems Rev C.01.07 SR2 [255] (Agilent Technologies).

Internal Standard

Internal standardization was used to compensate for analyte losses during sample preparation. Sodium succinate dibasic hexahydrate eluted between the peaks of AA and PA [s1]. The area ratio of the SCFAs and IS were used during the data processing to calculate the concentrations.

Validation of the Ultra-performance Liquid Chromatography Method

The quantitative method was validated in-house for each organic acid, by a set of parameters [i.e. limit of quantification (LOQ), the limit of detection (LOD), linearity, inter-assay precision, intra-assay precision, accuracy, selectivity, stability, and carry-over). The validation was carried out on a 2690 Alliance HPLC in combination with a 996 photodiode-array detector (PDA) (Waters, Milford, MA, USA).

Limit of Quantification (LOQ)

LOQ is the lowest amount of each SCFA, which can be quantitatively determined with suitable precision and accuracy. The LOQ, 0.25 mM for each SCFA, was determined by preparing the standard six times independently.

Limit of Detection (LOD)

The LOD is the LOQ divided by 3.3, which is the lowest amount that can be detected, but not necessarily quantified as an exact value. The 0.08 mM standard solution was prepared six times independently.

Linearity

The linearity of the method was evaluated by analyzing the calibrators, namely 0.25, 0.5, 1, 2.5, 5, 10, 15, and 25 mM. Each calibrator was prepared once and injected twice. After the calibration curve, the control sample was prepared in duplicated and each preparation was injected twice. The sequence was repeated three times.

Precision

For the precision the control sample was prepared dependently to test the repeatability of injection and independently to test the repeatability of sample preparation, each done in 6-fold.

Intra-assay Precision

Repeatability is the precision under the same operating conditions over a short interval of time. The intra-assay precision was carried out on two days.

Inter-assay Precision

The inter-assay precision expresses the within laboratories variation. For the intermediate precision on a different day, the results from the intra-assay precision during two days were combined.

Accuracy

The accuracy was carried out by determining the recovery of the spiked control sample and spiked 0.5 mM standard. Standard addition is done with a known amount at three levels (2.5, 5 and 10 mM). Samples were injected in duplicate.

Selectivity

Selectivity is defined as a degree to which a method can quantify the analyte in the presence of interferences. Therefore an injection of a blank, calibrator at 2.5 mM and each SCFA separately at a concentration of 2.5 mM were injected.

Stability

Stability of the control sample was tested in two ways. Stability was tested during 24-hours and different days. For the first test every 4 hours over 24-hours an injection of the control sample was done using the same vial. The second test was to test the stability of the control sample at RT and 8°C on different days. This test was carried out during four days.

Carry-over

After injecting the highest calibrator (25 mM), a blank was injected.

S2. Supplementary Results: Quantification of Short-chain fatty Acids in fecal suspension

Validation of the ultra-performance liquid chromatography method

Limit of quantification

The coefficient of variation (CV%) on the concentration of the 0.25 mM standard was below 10% and recovery was between 100 and 106%.

Limit of detection

The CV% on the concentration of the 0.08 mM standard was below 10%.

Linearity

The CV% on the concentration of each calibrator for each calibration curve was below 5%, except for the lowest calibrators (0.25 mM PA: 5.5%, 0.25 mM BA: 6.5%; 0.5 mM AA: 7% and 0.5 mM BA: 5.5%). The recovery of the concentration of each calibrator for each calibration curve was between 90 and 110%, except for the lowest calibrators (0.25 mM AA: 136%; 0.25 mM PA: 142%; 0.25 mM BA: 129%; 0.5 mM AA: 113%; 0.5 mM PA: 116%; 0.5mM BA: 112%). The relative retention time (RRT) of each calibrator for each calibration curve was between 0.97 and 1.03. the RRT was calculated against the mean retention time (Rt) at 2.5 mM. The coefficient of determination (r^2) was for each curve ≥ 0.9996. The overall CV% on the concentration for each SCFA present in the control sample was below 3%.

Precision

Intra-assay precision

The CV% on the concentration for each SCFA was below 3% and the CV% on the Rt for each SCFA was below 1% for both the dependently and independently prepared control samples for each day.

Inter-assay precision

The CV% on the concentration for each SCFA was below 6% and the CV% on RT for each SCFA was below 1% for both the dependently and independently prepared control samples.

Accuracy

The recoveries for each SCFA present in the control sample were between 93 and 101%, except when adding 10 mM standard the recoveries for PA and BA were between 99.75 and 89.49%. the recoveries for each SCFA present in the standard at the three spiking levels were between 93 and 101%.

Selectivity

No peaks were found in the elution range of the analytes.

Stability

When testing the stability of the control sample during a 24-hour period, the CV% on Rt was for each SCFA below 1% and on the concentration for each SCFA below 2%. Storage of the control sample at RT and 8°C during four days gave similar results. For both, the CV% on Rt was for each SCFA below 2% and CV% on concentration was for each SCFA below 4%.

Carry-over

No carry over at the retention times of the SCFAs was noticed.

Supplementary Tables

Table S1. Dilution scheme to obtain the calibrators.

Calibrator (mM)	Working solution (mM)	Volume working solution (µL)	Volume PBS (µL)
0.25	2.5	100	900
0.5	5	100	900
1	10	100	900
2.5	25	100	900
5	10	350	350
10	100	100	900
15	100	150	850
25	100	250	750

Table S2. Gradient programme for the analysis of short-chain fatty acids (SCFAs) in fecal water.

Time (min)	MP A (%)	MP B (%)	MP C (%)	Flow rate (mL/min)
0.0	100	0	0	1.0
1.0	100	0	0	1.0
15.0	80	10	10	1.0
16.0	0	50	50	1.0
26.0	0	50	50	1.0
27.0	100	0	0	1.0
35.0	100	0	0	1.0

MP: mobile phase.
